# Review: Sustainable Biosorbent and Biopolymeric Materials for Heavy Metal Adsorption—Advances, Challenges, and Perspectives

**DOI:** 10.3390/ma18204752

**Published:** 2025-10-16

**Authors:** André Lamounier Caixeta, Ana Carolina Nunes da Silva, Sarah Kalli Silva da Silva, Matheus de Carvalho Dias, Camila Monteiro Cholant, Tiago Moreno Volkmer, André Luiz Missio, Amanda Dantas de Oliveira, Mateus Meneguetti Ferrer, Yasir Anwar, Sabir Khan

**Affiliations:** Technological Development Center, Federal University of Pelotas, Pelotas 96010-610, Brazil; andre.caixeta@ufpel.edu.br (A.L.C.); acnsilva@ufpel.edu.br (A.C.N.d.S.); sarah.silva@ufpel.edu.br (S.K.S.d.S.); matheus.dias@ufpel.edu.br (M.d.C.D.); camila.cholant@ufpel.edu.br (C.M.C.); tiago.volkmer@ufpel.edu.br (T.M.V.); andre.missio@ufpel.edu.br (A.L.M.); adoliveira@ufpel.edu.br (A.D.d.O.); mmferrer@ufpel.edu.br (M.M.F.); yasir10188@gmail.com (Y.A.)

**Keywords:** adsorption, heavy metals, sustainable biosorbents, adsorption mechanisms, biopolymers

## Abstract

The contamination of water resources by heavy metals poses a serious environmental risk, and conventional treatment methods face significant limitations. This review addresses the issue by presenting a critical analysis of the development of sustainable biosorbent and biopolymeric materials for heavy metal adsorption, highlighting advances, challenges, and future perspectives. To this end, a systematic bibliometric analysis of 120 documents was conducted, extracted from the Scopus and Web of Science databases, covering the period from 2003 to 2025. The results indicate exponential growth in scientific interest in biopolymers such as cellulose, chitosan, lignin, and alginate, especially in the form of aerogels, which demonstrate high adsorptive capacity through mechanisms such as complexation, chelation, and ion exchange. The analysis also reveals the main factors influencing process efficiency, such as pH, temperature, and contact time. It is concluded that, although these sustainable materials are highly promising, challenges related to scalability, selectivity in complex effluents, and regenerability still need to be overcome to enable their large-scale industrial application, in line with the principles of the circular economy.

## 1. Introduction

Heavy metal contamination, including As, Pb, Hg, Cd, and Cr, poses a global environmental challenge, with serious impacts on human and animal health as well as ecosystem integrity. These elements, primarily released through industrial activities, mining, and agriculture, are toxic, persistent, and prone to bioaccumulation along food chains, causing effects such as oxidative stress, cellular damage, and chronic diseases [1,2].

In this context, it is essential to implement monitoring and remediation strategies, as well as to develop efficient, sustainable, and economically viable methods to mitigate contamination and protect vulnerable populations and the quality of natural resources.

According to [3], the lack of centralized national data on the subject makes it impossible to determine the exact number of affected individuals. However, data from UNICEF and WHO indicate that nearly 1 in 4 people still lack access to safe drinking water [4].

For the treatment of contaminated effluents, various technologies have been established. Conventional methods include chemical precipitation and ion exchange. Although effective under certain conditions, these techniques often face significant limitations, such as high implementation and operational costs, high energy consumption, low selectivity, and the generation of secondary waste, which requires additional treatment for disposal [1,5].

In this context, adsorption has emerged as a promising alternative, being an efficient, low-cost, and environmentally sustainable process, especially when utilizing adsorbent materials derived from biomass or agricultural waste. Such materials are abundant, renewable, possess active functional groups on their surface, and exhibit great potential for regeneration [6,7].

Polymeric membranes, produced from natural or synthetic polymers, are widely applied in sectors such as gas separation and, especially, in effluent treatment. The technical feasibility and sustainability of these materials stem partly from the affordable cost of their polymeric precursors. However, it is crucial to distinguish the material cost from the operational cost of the filtration process. While low-pressure systems like microfiltration (MF) and ultrafiltration (UF) are notable for their low energy demand, high-pressure methods such as reverse osmosis (RO) involve high power consumption, which significantly increases their application cost. This flexibility, combined with high separation efficiency, consolidates membranes as a strategic solution for water resource recovery [8,9,10,11].

The valorization of agro-industrial waste for the formulation of new materials directly aligns with the principles of the circular economy and Sustainable Development Goals (SDGs) 9, 12, and 13. A notable application of this approach is the fabrication of polymeric membranes, which, although part of filtration systems that can have high operational costs (such as RO), represent an advancement when produced from sustainable and low-cost polymers, thereby optimizing separation efficiency and technical feasibility [11,12,13].

Given the need for integrative analyses in this field, this review provides a critical and comparative assessment of sustainable biosorbent and biopolymeric materials applied to the adsorption of heavy metals. The work is structured to address the main advances in the development of different material forms, including the fabrication of adsorptive membranes and the formulation of aerogels and hydrogels, all based on biopolymers such as cellulose, chitosan, lignin, and alginate. Additionally, it discusses the cross-cutting challenges of selectivity in complex effluents, regenerability, and industrial scalability; and finally, presents future perspectives for the practical application of these technologies. The aim is to highlight that, although laboratory efficiency is high, commercial viability depends on overcoming economic bottlenecks and standardizing synthesis routes from waste materials, thus consolidating a solution aligned with the circular economy.

## 2. Database

Bibliometric analyses allow for the quantitative and statistical evaluation of scientific production, identifying patterns, trends, and indicators such as annual publication output, the most productive countries, co-authorship networks, and the most frequently used keywords.

A specific bibliometric search focused on the biopolymers worked on in this review was performed to identify studies evaluating the use of lignin, cellulose, chitosan, and alginate as adsorbents for the removal of heavy metals from water. Searches were conducted in two major scientific databases, Scopus and the Web of Science Core Collection (Clarivate Analytics/Thomson Reuters), using literal search terms with field-specific tags.

For Scopus, the TITLE-ABS-KEY field was used, while for Web of Science the Topic field (TS) was applied. The search strings were designed to capture relevant studies, for example: TITLE-ABS-KEY(“lignin”) AND TITLE-ABS-KEY (“adsorbent” AND “adsorption”) AND TITLE-ABS-KEY (“heavy metal”) AND TITLE-ABS-KEY (“water contamination”) for lignin; similar structured queries were used for chitosan, cellulose, and alginate. Searches were conducted using English keywords, without restrictions on the language of publication. Searches covered the period from 2003 in Scopus and from 2003 in Web of Science until the first quarter of 2025, reflecting the maximum time coverage of each database.

All retrieved records were exported in CSV format from Scopus and BibTeX format from Web of Science. Each record contained author names, article title, source, publication year, DOI, abstract, and keywords. Records were imported into R—4.5.1 [14] on ambient RStudio (Boston, Ma, USA) [15] using the bibliometrix package for reproducible bibliometric analysis.

A two-step deduplication procedure was applied, prioritizing DOI-based removal of duplicates followed by title-based normalization to account for capitalization, punctuation, and spacing differences. Prior to deduplication, a total of 140 records were identified, comprising 112 records from Scopus (lignin: 8; cellulose: 44; chitosan: 44; alginate: 16) and 28 records from Web of Science (lignin: 2; cellulose: 13; chitosan: 9; alginate: 4). After removal of 20 duplicate records, 120 unique publications remained for analysis.

The study selection process was documented following the PRISMA 2020 Statement guidelines for transparency in systematic reviews. Although no quantitative synthesis (meta-analysis) was performed, the PRISMA flow diagram was applied to illustrate the identification, screening, and inclusion of bibliographic records. All search outputs from Scopus and Web of Science were merged, and duplicates were removed using a two-step procedure (DOI-based, followed by normalized titles).

**Figure 1:**
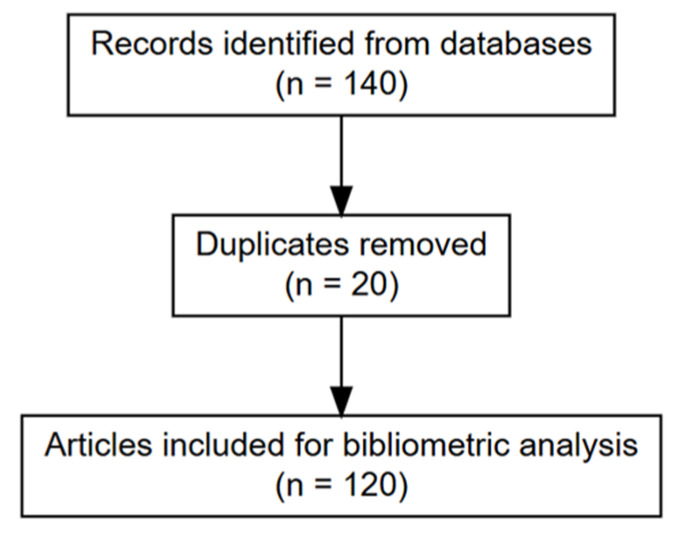
Data flow, showing duplicate removal and final unique records included.

A total of 140 records were initially retrieved, of which 20 duplicates were removed, resulting in a final corpus of 120 unique publications included in the bibliometric assessment, as illustrated in Figure 1. All raw datasets (CSV files) and R scripts are provided as supplementary material to ensure reproducibility and transparency. The PRISMA 2020 framework was applied as a methodological guide to document the selection process and enhance reporting clarity, rather than as an analytical tool (e.g., meta or metafor packages for effect size aggregation).

## 3. Adsorption of Heavy Metals

Heavy metals such as lead, Cd, Hg, and Cr are persistent, toxic, and non-biodegradable pollutants that pose serious risks to the environment and human health. According to studies by [16,17], they can be released naturally through geological processes such as rock weathering and volcanic eruptions, or through anthropogenic activities, including industrial operations, agriculture, and improper disposal of waste and urban effluents, leading to soil and water contamination, bioaccumulation in food chains, and toxicity to aquatic and terrestrial organisms, as well as adverse human health effects, including neurological, renal, and hepatic damage, cancer, and developmental disorders [18,19].

In accordance with [18,20], to mitigate these impacts, various removal methods have been developed: adsorption, using materials such as activated carbon, clays, MOFs, and biosorbents, is highly efficient, low-cost, and regenerable; membrane filtration provides near-complete removal but is costly and prone to fouling; chemical precipitation is simple and inexpensive but generates sludge; ion exchange is selective and efficient, though more expensive; biological methods, such as biosorption and bioaccumulation, are sustainable and low-cost but slower; and electrochemical and photocatalytic techniques, including electrocoagulation and TiO_2_-based processes, show great potential, yet remain scarcely applied at industrial scale, as stated by [18,20,21,22,23,24].

### 3.1. Factors Influencing the Bioavailability of Heavy Metals

Several factors govern the bioavailability of heavy metals, defined as the extent to which they can be absorbed by organisms or participate in biological processes. The factors affecting bioavailability can be clearly grouped into three main categories: environmental factors (e.g., temperature, contact time, adsorption), chemical factors (e.g., solubility, pH, complexation, interference from other ions), and biological factors (e.g., organism uptake, metabolic activity, microbial interactions). Understanding these categories is essential for predicting the mobility, fate, and potential risks of heavy metals in contaminated environments, according to [25,26].

### 3.2. Physicochemical Mechanism of Adsorption

**Figure 2:**
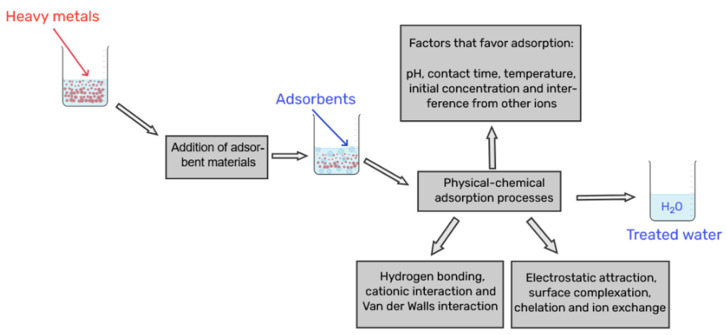
General processes of adsorption and removal of heavy metals present in contaminated effluents.

The removal of heavy metals from contaminated effluents occurs through various physicochemical interactions, and the efficiency and selectivity of these mechanisms depend on the material’s properties, such as surface area, porosity, and the presence of functional groups [27,28,29], For instance, in materials like aerogels, the nanoporous structure provides a vast surface area for adsorption. In hydrogels, performance is governed by the swelling of the polymer network and the diffusion of metal ions to internal active sites, such as amino groups [30]. Analogously, advanced membrane techniques, such as Polymer-Enhanced Ultrafiltration (PEUF), combine the physical separation of the membrane with the chemical capture of ions by polymers with specific functional groups [18]. This process increases the overall efficiency and selectivity of heavy metal removal [31,32], as illustrated in Figure 2, and can be summarized by the following mechanisms:

### 3.3. Physical Adsorption

Physical adsorption occurs rapidly and is low-cost, offering excellent cost-effectiveness; however, it presents lower selectivity compared to chemical adsorption. The adsorption mechanisms can be classified based on the type of interaction involved: van der Waals forces, electrostatic interactions (including cationic interactions), or ion exchange [28,33,34]. The predominance of each mechanism depends on the type of metal ion, the pH of the solution, and the functional groups present on the adsorbent’s surface. When combining porosity and functionality, porous materials are optimized for efficient removal of heavy metals from water [28,32,33].

The behavior of metal ions such as Pb^2+^, Cd^2+^, and Hg^2+^ in aqueous media involves their diffusion through the pores of the aerogel (based on silica, carbon, or polymers). This diffusion leads to their deposition onto the internal surfaces of the porous adsorbent through van der Waals forces, electrostatic interactions, and/or ion exchange, depending on the specific metal and functional groups present [29,35]. As a result, the large surface area of the porous structure provides numerous active sites for the metal ions to physically and/or electrostatically attach [31,32]. The high porosity and interconnected pore network act as nanoscale “traps,” enhancing ion diffusion and retention and increasing overall adsorption capacity.

The greater the specific surface area, the more adsorption sites are available, as the pores act as traps where the ions are retained by physical processes due to nanoscale confinement.

The efficiency of physical adsorption is influenced by factors such as pH, ion size, electronegativity, and hydration radius of the metal ions. Metals like Pb^2+^ typically exhibit higher affinity due to their greater electronegativity and smaller hydration radius, resulting in stronger electrostatic interactions.

Thus, the use becomes effective because aerogels undergo supercritical drying, which results in the preservation of their porous structure. However, physical adsorption presents a relatively weaker interaction between the product surface (aerogel) and heavy metals.

#### Hydrogen Bonding

This is an important but secondary intermolecular interaction in adsorption when adsorbents have functional groups such as -OH, -NH_2_, or -COOH [29]. This interaction is particularly significant in hydrogels, where the high capacity for water absorption is governed by extensive hydrogen bonding between the polymer’s functional groups (-OH and -NH_2_) and water molecules, which stabilizes the swollen structure [30]. Hydrogen bonding is generally considered a type of physical adsorption (physisorption), as it involves relatively weak interactions compared with covalent or ionic bonds typical of chemical adsorption (chemisorption) [29]. When applied to the removal of heavy metals from water, this interaction can indirectly contribute to the efficiency of the adsorbent, such as an aerogel [29]. The bond can be disrupted by changes in pH or competition with water molecules, acting synergistically with van der Waals forces and electrostatic interactions to enhance overall adsorption efficiency [29].

### 3.4. Chemical Adsorption

#### 3.4.1. Adsorption Mechanism

Chemical adsorption, or chemisorption, involves stronger and more specific interactions than physisorption, resulting in the formation of chemical bonds between the adsorbate and the adsorbent surface [36]. The primary mechanisms in biosorbents include surface complexation, chelation, and ion exchange, which are considered chemical interactions due to the formation of coordination or covalent bonds with functional groups.

#### 3.4.2. Electrostatic Attraction

Electrostatic attraction, while sometimes grouped with chemical mechanisms, is mainly a physical interaction when it occurs through the simple attraction of oppositely charged ions without bond formation [29]. The surface charge of biosorbents is intrinsically dependent on the pH of the medium, which determines which ions will be attracted.

For example, at pH levels where functional groups like carboxyl (-COOH) and hydroxyl (-OH) are deprotonated (-COO^−^, -O^−^), the surface becomes negative, attracting metal cations such as Pb^2+^, Cd^2+^, and Cu^2+^. Conversely, at acidic pHs, groups like amino (-NH_2_) can be protonated (-NH_3_^+^), creating positive sites that attract metal anions, such as CrO_4_^2−^ and AsO_4_^3−^ [34].

Specific cases such as cation–π interactions in carbon-based aerogels contribute to adsorption but may also have a partial chemical character [16]. However, the term can also refer to aerogels derived from synthetic precursors (e.g., resorcinol-formaldehyde), which do not fall under the definition of a biosorbent [37].

#### 3.4.3. Surface Complexation

Surface complexation is an adsorption mechanism in which metal ions form coordinated complexes with functional groups present on the surface of the adsorbent, mainly in functionalized porous materials such as carbon or silica aerogels, biopolymeric hydrogels, and membranes used in complexation-ultrafiltration processes [30,38]. Silica aerogels are inorganic materials formed from a silicon dioxide network [39], while carbon aerogels are typically derived from the pyrolysis of organic precursors [40]. These materials have active sites such as -OH, -COOH, and -NH_2_, which can donate electron pairs to heavy metals such as Pb^2+^, Cd^2+^, and Cu^2+^, forming coordination bonds and thereby engaging in chemical adsorption through partial or full sharing of electron pairs to stabilize the metal ion on the surface [30,31,38,41]. This process can be used both to remove heavy metals from water and to mobilize them, depending on the characteristics of the complexing agent and the solution conditions [22].

Its mechanism occurs mainly through coordination with oxygenated groups, where transition metals such as Cu^2+^ or Hg^2+^ bind to carboxylic (-COOH) or hydroxyl (-OH) groups, forming structures of the type M^2+^-O-R [31].

The effect of pH alteration <4, the protonation of the groups reduces complexation, while at neutral/alkaline pH (5–9), deprotonation favors metal-ligand binding. Finally, the selectivity for metals shows high affinity for hard ligands (Fe^3+^, Al^3+^) or soft ligands (Hg^2+^, Ag^+^), which can result in their selective capture [31,41,42].

On the other hand, the effectiveness of physical adsorption in aerogels is intrinsically linked to their highly porous structure [43]. This structure, characterized by a vast internal surface area, is preserved through specialized drying processes such as supercritical drying or lyophilization. Unlike conventional drying, which causes pore collapse (and loss of surface area), these methods remove the solvent from the gel without destroying the nanoporous network, thereby ensuring the availability of numerous sites for physical adsorption [44].

#### 3.4.4. Chelation

Chelation is a specific form of chemical adsorption, where a metal ion is surrounded by multiple functional groups from the adsorbent, forming a stable cyclic structure.

According to studies by [27,28], the chelation process is a specific case of complexation in which a metal ion is surrounded by multiple functional groups from the adsorbent, forming a highly stable cyclic structure. This mechanism is critical for the removal of highly toxic metals, as is the case with metal cations such as Cd^2+^ and Hg^2+^. Among its characteristics, groups such as EDTA (-N(CH_2_COO^−^)_4_), dithizones, or thioureas act as “molecular claws,” functioning as polydentate ligands in this system. They exhibit a high stability constant of formation (Kf), as seen in the Hg^2+^-EDTA complex (log Kf ≈ 21.5). The use of aerogels functionalized with amino (-NH_2_) or thiol (-SH) groups has demonstrated high efficiency for the chelation of Hg^2+^ [45]. Furthermore, it is important to note that the desorption process requires strong eluents, such as 1 M HNO_3_, which may degrade the material after repeated cycles.

#### 3.4.5. Ion Exchange

In ion exchange is also a chemical mechanism, metal ions in solution are captured by substituting lighter cations (e.g., H^+^, Na^+^), that is, by ions with similar charges previously bound to a given solid material [46]. The metal ions present in contaminated water are attracted to functional groups and replace the less desirable ions. This is a reversible process that relies on bonding interactions between the ions and the surface functional groups, with the equilibrium of the solution affected by ion concentration, selectivity, and pH, distinguishing it from purely physical adsorption [46,47].

The presence of competing ions in real wastewater reduces the removal efficiency of heavy metals, especially for conventional adsorbents. Advanced and selective materials, such as MOFs and nanocomposites, show better performance but still face challenges in complex environments. Testing under real conditions is essential for validation prior to large-scale application [18,48].

This mechanism is dominant in aerogels with charged groups such as -SO_3_^−^ and -COO^−^ [49] Its fundamental principle lies in the exchange capacity, which is directly influenced by the density of functional groups present in the aerogel [49]. Regarding selectivity, it is known that this process is influenced by the charge and radius of the ion. Regeneration is a crucial step in ion exchange, in which acidic (e.g., HCl) or saline (e.g., NaCl) solutions are used to restore the active sites of the aerogel [50].

#### 3.4.6. Factors Affecting Adsorption

##### pH

The pH factor directly influences adsorption through the protonation and deprotonation of surface groups. At low pH (acidic medium), competition with H^+^ can reduce adsorption, whereas at neutral/alkaline pH, deprotonation of -OH or -COOH groups is promoted, favoring the binding with metal cations [27,29].

##### Contact Time

Research such as that reported in [27,29,51] discuss contact time as a critical parameter in the adsorption of heavy metals by aerogels, due to its direct influence on process kinetics and maximum removal capacity. These authors highlight that the process can be divided into three phases: (1) diffusion of ions to the aerogel surface, occurring rapidly; (2) adsorption at active sites, which is controlled by surface chemistry; and (3) filling of internal pores, which occurs more slowly and varies according to porosity.

##### Temperature

Temperature is a critical parameter that influences both the kinetics and equilibrium of adsorption. The thermodynamic nature of the process can be endothermic (favored by an increase in temperature) or exothermic (disfavored by an increase in temperature), depending specifically on the adsorbent-adsorbate system. Generally, physisorption is an exothermic process, whereas chemisorption can be endothermic, requiring energy to activate bond formation [27].

An example of this complexity is observed in studies with cellulose aerogels, such as that by [41]. In that study, the adsorption capacity for metal ions initially increased with temperature, a behavior attributed to the activation of more adsorption sites. However, after reaching an optimal temperature, the capacity began to decrease, indicating that the predominantly exothermic nature of the process then governed the equilibrium. This demonstrates that generalization is not appropriate and that temperature optimization is essential to maximize the removal efficiency of heavy metals for each specific system.

##### Initial Concentration

Previous studies such as [52,53] highlight that the initial concentration of heavy metals in solution is an important factor affecting adsorption efficiency. The higher the initial concentration, the greater the amount of metal that can generally be adsorbed until the adsorbent reaches saturation.

However, the adsorption rate and removal capacity can also be influenced by this concentration, as high concentrations may lead to rapid exhaustion of adsorption sites and potentially reduce process efficiency if the system is not properly optimized.

Additionally, different adsorption kinetic models consider the initial concentration to determine the process rate and the maximum number of metals the adsorbent can retain. Thus, initial concentration is a crucial parameter in optimizing the adsorption process to ensure maximum removal of heavy metals from aqueous solutions.

##### Interference from Other Ions

The presence of competing ions, such as Na^+^, Ca^2+^, and Mg^2+^, as well as anions like Cl^−^ and SO_4_^2−^—for example, in industrial effluent environments—can compromise the adsorption efficiency of target metals [27]. Among these, divalent cations (Ca^2+^, Mg^2+^) exert greater interference than monovalent ones (Na^+^, K^+^) due to their higher charge, resulting in more intense competition for the adsorbent’s active sites.

According to [52], the adsorption efficiency of heavy metals by materials such as fly ash and conducting polymers is directly affected by the presence of other ions in solutions. Among the determining factors, pH stands out as one of the most relevant, as it influences both the speciation of metals and the surface charge of the adsorbent, modifying its affinity for different competing ions. Furthermore, the concentration of metals and the pH of the medium affect the selectivity of the adsorbent, making it possible for the removal capacity of a given metal to decrease due to competition for adsorption sites.

Therefore, it is essential to consider the presence of other ions when analyzing adsorption performance, as their interference can alter the chemical conditions of the system and consequently reduce the efficiency of the heavy metal removal process.

## 4. Sustainable Materials and Technologies for Water Purification

### 4.1. Biopolymers as Adsorbents

As previously reported in this work, adsorption by adsorbent materials stands out as one of the most effective methods for removing metal ions from aqueous solutions, due to its operational simplicity, low cost, and high efficiency [54]. It is also noteworthy that the advantages of this process are significantly enhanced when the adsorbent has regeneration potential and can be properly disposed of after use [55].

In this context, the choice of adsorbent material becomes an essential factor for process efficiency [56]. Among the different classes of materials, biopolymers have received increasing attention due to their abundance, biodegradability, adjustable chemical structure, and renewable origin. These sustainable polymers can be obtained from agro-industrial residues, such as sugarcane bagasse, rice husks, and fruit and vegetable waste [57], for instance, lemon waste used to remove phosphate [58] or from fishery industry residues, such as crustacean shells [59], promoting the utilization of by-products and the development of environmentally responsible technologies.

Biopolymers have been applied in various stages of wastewater treatment, including coagulation and flocculation, sludge dewatering, chemical contaminant removal, membrane fouling control, and advanced processes such as oxidation and adsorption, although most applications are still restricted to the laboratory scale [60]. Among the polymeric adsorbents, those derived from carbohydrates, such as cellulose, chitosan, lignin, and alginate, stand out. These materials combine environmental sustainability with functional properties that favor the adsorption of metal ions.

Such polymers have high surface area, structural variety, and active functional groups, such as amines, hydroxyls, and phenolic hydroxyls. These groups promote physicochemical interactions with metal ions, forming stable complexes through chelation mechanisms, conferring high hydrophilicity and efficiency in the treatment of wastewater contaminated by heavy metals [61,62].

Below, we will detail some of the most commonly used natural biopolymers as adsorbents—cellulose, chitosan, lignin, and alginate—highlighting their relevant properties, origins, modes of interaction with metal ions, and sustainable applications in wastewater treatment.

#### 4.1.1. Cellulose

**Figure 3:**
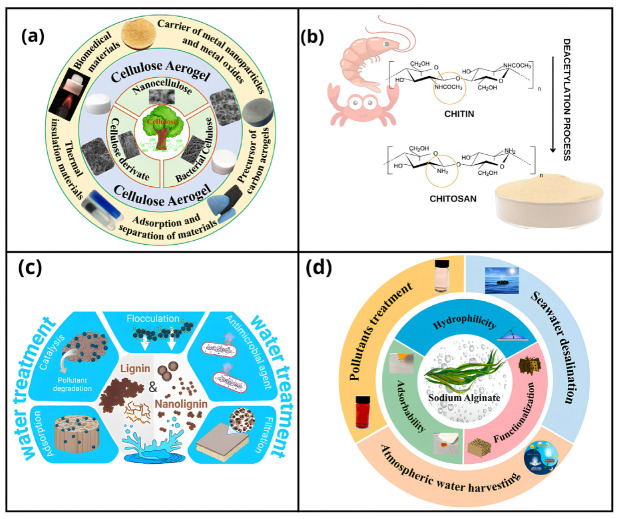
Schematic representation adapted from the sources, structures, and applications of natural biopolymers: (**a**) cellulose, reproduced from [66], Ma, Y.; Hu, Y.; Yang, X.; Shang, Q.; Huang, Q.; Hu, L.; Jia, P.; Zhou, Y. Fabrication, Functionalization and Applications of Cellulose Based Aerogels: A Review. International Journal of Biological Macromolecules 2025, 284, 138114. Copyright Elsevier 2025. (**b**) chitosan, reproduced from [69], Gonçalves, J.O.; Strieder, M.M.; Silva, L.F.O.; dos Reis, G.S.; Dotto, G.L. Advanced Technologies in Water Treatment: Chitosan and Its Modifications as Effective Agents in the Adsorption of Contaminants. International Journal of Biological Macromolecules 2024, 270, 132307. Copyright Elsevier 2025. (**c**) lignin and nanolignin, reproduced from [74], Camargos, C.H.M.; Yang, L.; Jackson, J.C.; Tanganini, I.C.; Francisco, K.R.; Ceccato-Antonini, S.R.; Rezende, C.A.; Faria, A.F. Lignin and Nanolignin: Next-Generation Sustainable Materials for Water Treatment. ACS Appl. Bio Mater. 2025, 8, 2632–2673, under the CC BY 4.0; and (**d**) sodium alginate, reproduced from [32], Zheng, D.; Wang, K.; Bai, B. A Critical Review of Sodium Alginate-Based Composites in Water Treatment. Carbohydrate Polymers 2024, 331, 121850. Copyright Elsevier 2025.

Cellulose is the most abundant natural polysaccharide on the planet, being the main structural component of plant cell walls. It is a linear homopolymer composed of repeating β-D-anhydroglucopyranose units linked by β-1,4 glycosidic bonds [63]. Its wide availability from sources such as trees, plants, algae, and agro-industrial residues—such as corn straw and rice husk—and textiles [64,65], makes it a promising and economically viable precursor to produce adsorbent materials. As illustrated in Figure 3a, different forms of cellulose have been explored in the manufacture of functional aerogels, applied in areas ranging from controlled release systems to thermal insulation and adsorption of environmental contaminants [66].

In this context, ref. [67] demonstrated the application of a mold made from recycled cellulose, obtained from used paper, as a support for the fabrication of a cobalt nanosensor (SNC) functionalized with 1-(2-hydroxy-1-naphthylazo)-2-naphthol-4-sulfonic acid (HNNSA). The resulting material showed high sensitivity and selectivity in the detection and removal of Co (II) ions, with a detection limit as low as 1.13 × 10^−7^ M. The porous structure of the cellulosic matrix, confirmed by techniques such as XRD, SEM, TEM, and nitrogen adsorption analysis, was fundamental to the efficiency. Other researchers have also demonstrated the versatility of cellulose-based matrices in various environmental remediation contexts. For example, ref. [68] developed a hybrid aerogel composed of a metal–organic framework modified with ethylenediaminetetraacetic acid (UiO-66-EDTA) incorporated into a matrix of cellulose nanofibers (CNF) and carboxymethyl cellulose (CMC) for applications in the remediation of wastewater containing heavy metals. In the study, nine metal ions (Cr^3+^, Cu^2+^, Co^2+^, Ni^2+^, Mn^2+^, Zn^2+^, Sn^4+^, Fe^3+^, and Zr^4+^) were tested, and the U-EDTA CCA aerogel exhibited excellent adsorption capacity, achieving up to 98% removal for the most efficiently adsorbed metal ion and maintaining efficiency above 91% in mixed solutions containing five types of metal ions. The superior performance compared to pure CNF aerogels was attributed to the strong chelation between the target ions and EDTA, combined with the micrometer-scale porosity and anisotropic structure of the aerogel, which enhanced the contact between active sites and metal ions, rendering the material highly effective and reusable for the removal of multiple heavy metals.

#### 4.1.2. Chitosan

Chitin is the second most abundant biopolymer in nature, found mainly in the exoskeleton of crustaceans, insects, and in the cell walls of fungi. Through its partial deacetylation, chitosan is obtained—a linear polysaccharide that combines structural and functional characteristics making it highly promising for adsorption applications. As illustrated in Figure 3b, this conversion process involves the removal of acetyl groups from chitin, resulting in a structure rich in free amine groups, which are highly reactive and fundamental for the complexation of metal ions in aqueous solutions [69]. Its main sources include crustacean shells and residues from fungi and insects, contributing to its profile as a renewable, accessible resource with strong environmental appeal [70]. The presence of primary amine functional groups in its structure favors complexation with metal ions through electrostatic interactions, increasing its efficiency in contaminant removal processes in aqueous media [71]. Recently, this biopolymer has received growing attention as a basis for the formulation of sustainable adsorbents, due to its biodegradability, economic viability, and abundance in organic waste [72].

A study conducted by [73] demonstrates the potential of chitosan in its nanostructured form, by coating cotton fibers with nano chitosan (NCCF) crosslinked with citric acid for the removal of heavy metals from industrial effluents. The modification provided the material with greater surface area and porosity, resulting in adsorption capacities superior to those of the natural fiber. The tests revealed maximum adsorption values, according to the Langmuir model, of 4.76 mmol/g for Cd^2+^, 6.40 mmol/g for Pb^2+^, and 12.50 mmol/g for Cr^6+^. This work reinforces the feasibility of nanostructured chitosan as an efficient and sustainable material for industrial applications in the treatment of water contaminated with heavy metals.

In the study conducted by [30], an adsorbent nanocomposite was developed from chitosan extracted from shrimp shell waste, a widely available byproduct of the fishing industry. The resulting CS-TPP-NSi nanocomposite demonstrated outstanding adsorptive performance in the removal of heavy metals, with maximum capacities of 112.35 mg/g for lead and 60.97 mg/g for Zn, according to the Langmuir model. In addition to its high capacity, the material exhibited rapid adsorption kinetics and good efficiency at different pollutant concentrations, demonstrating versatility and high potential for real-world applications in the treatment of contaminated water.

#### 4.1.3. Lignin

Unlike linear polysaccharides such as cellulose and chitosan, lignin is an amorphous aromatic polymer with a complex three-dimensional structure formed by phenylpropane units—guaiacyl (G), syringyl (S), and p-hydroxyphenyl (H). As shown in Figure 3c, this structural configuration gives lignin and nanolignin properties such as high rigidity, thermal stability, and chemical resistance, making it a promising matrix for application in functional materials [74]. From a sustainability perspective, one of the most relevant aspects of lignin is its origin: it is a large-scale byproduct of the pulp and paper industries and bioethanol production, generally underutilized as a low-value energy source or simply discarded. In this sense, its valorization as a precursor for high-performance adsorbent materials represents a clear example of the circular economy, promoting the conversion of industrial waste into technological solutions for environmental remediation.

Furthermore, lignin stands out as a low-cost adsorbent compared to conventional materials such as activated carbon, especially in the removal of toxic metal ions. Due to the presence of polyhydric phenols and additional functional groups on its surface, it exhibits high adsorptive efficacy, which favors interactions with contaminant species and increases the retention capacity of metals in solution [75].

#### 4.1.4. Alginate

Alginate is a linear anionic polysaccharide extracted from brown algae such as *Ascophyllum nodosum*, *Macrocystis pyrifera*, and *Laminaria hyperborea* [76], making it a renewable marine resource with low environmental impact, as shown in Figure 3d. Its structure is composed of β-D-mannuronic acid (M) and α-L-guluronic acid (G) units, whose proportion and distribution directly influence its physicochemical properties. It stands out for its ability to form hydrogels through ionic interactions between G-blocks and multivalent cations such as Ca^2+^ and Al^3+^, resulting in a three-dimensional network widely used in adsorption processes [77]. While monovalent salts such as Na^+^ and K^+^ generate viscous solutions, divalent and trivalent cations induce the formation of insoluble gels, which reinforces their applicability in effluent treatment systems [78]. Additionally, alginate contains hydroxyl and carbonyl groups in its chain, which can be chemically modified through techniques such as crosslinking or surface grafting to further enhance its adsorptive capacity [79].

In this context, [80] proposed the synthesis of an advanced composite based on crosslinked alginate with rice husk ash, graphene oxide, and chitosan nanoparticles (CL-ARCG-CNP), aiming for the efficient removal of Pb^2+^ ions in aqueous systems. The material exhibited favorable morphological and structural properties, as well as a surface area of approximately 148.44 m^2^/g. It achieved an adsorptive capacity of 242.5 mg/g and a removal efficiency of 95.2% after 240 min of contact. Furthermore, reusability tests showed stable performance for up to five consecutive cycles. These results reinforce the feasibility of modified alginate as a functional platform for the adsorption of heavy metals in wastewater.

#### 4.1.5. Sustainable Aerogels

Aerogels are highly porous solid materials with a three-dimensional structure characterized by extremely low density, ranging from 0.0011 to 0.5 g/cm^3^, and porosity between 90% and 99% [81]. As described by [82], and later reinforced by [83], the formation of aerogels occurs by replacing the liquid in a gel with a gaseous phase, resulting in a network of nanometric pores. Additionally, these materials exhibit a very large specific surface area, which can reach values between 10 and 2000 m^2^/g [84]. This structural combination gives aerogels remarkable efficiency in capturing contaminants in aqueous media, favored by the high surface area and highly organized pore network, which enhance adsorption processes [85]. Due to these properties, aerogels have attracted increasing interest in the scientific community, especially for their ability to establish effective adsorptive interactions with heavy metals and other pollutants [86].

According to the study by [87] who developed a sustainable aerogel from mandarin peel, a promising alternative was proposed for dye removal and the valorization of agro-industrial waste. The process involved purifying the biomass with NaOH to remove non-cellulosic components, followed by modification with Tween 80 to increase porosity and lyophilization. Gelation was achieved without the use of conventional solvents, using only pectin and NaOH. The resulting aerogel exhibited remarkable porosity (88% of the mesopore volume), a specific surface area of approximately 154 m^2^/g, and an average adsorption capacity of 79 mg·g^−1^ of methyl orange in aqueous solution. Due to its high porosity, natural origin, and remarkable adsorption capacity, the material shows strong potential for use in pollutant remediation systems, gas capture processes, and broader environmental protection applications.

In addition to being economical, environmentally friendly, and efficient in producing a good adsorbent product with ideal porosity, it proved to be a new opportunity for the application of value-added and environmentally sustainable materials through the economic recycling of food by-products.

Given the above, the pursuit of even greater performance and multifunctional solutions has driven the synergistic combination of biopolymers with other advanced technologies. Instead of acting only as dispersed adsorbents, these sustainable materials can be incorporated as active components in engineering matrices, such as filtration membranes. This hybrid approach not only leverages the high surface area and functional groups of biopolymers but also integrates them into a robust physical separation process, giving rise to a new class of high-performance composite materials.

### 4.2. Purification Techniques

#### 4.2.1. Filtration and Membrane Separation

**Figure 4:**
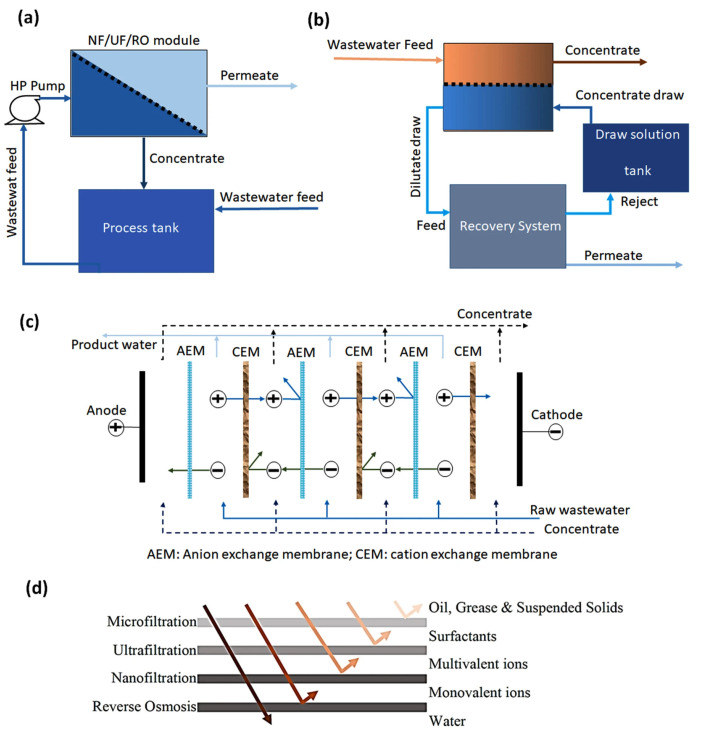
Different types of membrane filtration techniques: (**a**) nanofiltration, ultrafiltration, or reverse osmosis method; (**b**) forward osmosis process; (**c**) electrodialysis method in which alternative charged positive and negative membranes take place; and (**d**) the separation capabilities of different membranes against different pollutants. Reproduced from [18], Qasem, N.A.A.; Mohammed, R.H.; Lawal, D.U. Removal of Heavy Metal Ions from Wastewater: A Comprehensive and Critical Review. npj Clean Water 2021, 4, 36, under the CC BY 4.0.

Membrane-based techniques for filtration and separation of metal ions have gained prominence in wastewater treatment, especially due to their efficiency in removing heavy metals [18]. These technologies include nanofiltration, forward osmosis, reverse osmosis, electrodialysis, microfiltration, and ultrafiltration, and are classified according to their different filtration techniques as represented in Figure 4a–c, membrane pore size, operating pressure, solution concentration, and the size of the particles or ions to be removed, as illustrated in Figure 4d [18,38]. Membranes have demonstrated high efficacy in retaining metal ions such as Cd^2+^, Pb^2+^, Ni^2+^, Cu^2+^, Al^3+^, Co^2+^, Zn^2+^, Mn^2+^, and Cr^6+^, contributing significantly to the treatment of contaminated waters [88].

Despite the high technical efficiency of membrane filtration processes, a complete assessment of their sustainability requires an analysis of their entire life cycle. In this context, Life Cycle Assessment (LCA) is a important tool for quantifying environmental impacts, such as CO_2_ emissions.

Comparative analyses indicate that the main source of emissions for polymeric membranes operating under pressure (such as UF/RO) occurs during the operational phase, due to the high electricity consumption of the pumps. In contrast, for sustainable adsorbents like aerogels and hydrogels, the greatest environmental impacts are concentrated in the production phase, stemming from the energy-intensive drying processes and the use of solvents. Thus, the choice between these technologies involves a fundamental trade-off: the operational energy impact of membranes versus the production energy and materials impact of adsorbents. This environmental cost–benefit perspective is vital when considering large-scale applications [89,90].

In addition to life cycle analysis, a fundamental practical challenge for large-scale application is the performance of membranes in real wastewater, which is often hampered by fouling and scaling. The presence of a complex ionic matrix and organic matter in industrial effluents leads to the clogging and degradation of membranes, a major drawback for systems like Reverse Osmosis. This phenomenon not only reduces removal efficiency but also necessitates pre-treatment steps and periodic cleaning, which incur additional operational and energy costs. The literature highlights a clear knowledge gap in this area, as most studies utilize synthetic wastewater, which does not represent the complexity of real effluents. Therefore, the successful transition of these technologies from the laboratory to industry critically depends on the development of new membranes with enhanced anti-fouling properties and the validation of their performance under realistic operating conditions [18].

#### 4.2.2. Nanofiltration

Nanofiltration (NF) is a pressure-driven separation technique that uses semi-permeable membranes with pore sizes ranging from 0.5 to 10 nm, thus operating between ultrafiltration and reverse osmosis technologies [91]. This characteristic allows NF to retain divalent ions such as Mg^2+^, Ca^2+^, Pb^2+^, Co^2+^, Mn^2+^, and Zn^2+^, making it effective in the purification of aqueous solutions. It is also notable for enabling the relatively simple and large-scale production of membranes. The main manufacturing methods for these membranes include interfacial polymerization and phase inversion, techniques widely employed in industry due to their efficiency and technical feasibility [92].

The search for NF membranes with greater selectivity, permeability, and durability has driven the development of new technologies. In this context, ref. [93] detailed the advancement of thin-film nanocomposite (TFN) membranes, which have emerged as an evolution of the conventional thin-film composite (TFC) architecture, widely used in NF, FO, and RO membranes). The TFC structure, which consists of a selective polyamide (PA) layer over a porous support, presents challenges such as susceptibility to fouling and a trade-off between permeability and selectivity. TFN technology overcomes these limitations by incorporating nanofillers into the selective PA layer during its formation. This approach enhances membrane performance, positioning TFN membranes for nanofiltration as a next-generation solution for the efficient removal of heavy metal ions from water [93].

#### 4.2.3. Microfiltration

Microfiltration is a separation technique that uses microporous membranes capable of retaining particles with sizes between 0.1 and 10 μm, operating under low pressure. Its application is quite diverse, being used in the treatment of solvents, fluids, and solutions for the removal of micrometric particles such as bacteria, protozoa, viruses, pollutants, and other contaminants [18]. By operating similarly to conventional filtration but with greater precision, MF is especially suitable for the removal of suspended matter. The process is driven by a static pressure difference, while separation occurs predominantly by a sieving mechanism. In general, microfiltration is preferably applied for the retention of dissolved solids and macromolecular organic compounds [94].

In their study, ref. [95] developed innovative microfiltration membranes composed of tin oxide (SnO_2_) dispersed in a polyvinyl chloride (PVC) matrix, aiming to separate oil-in-water emulsions. Using the phase inversion method, the authors produced membranes with different nanoparticle concentrations, highlighting the formulation with 1 wt% SnO_2_, which showed rejection rates of up to 99.6% for chemical oxygen demand (COD). This superior performance was attributed to the higher porosity and reduced hydrophobicity of the membrane, as well as the negative surface charge promoted by the nanoparticles, which intensified the repulsion between oily contaminants and the membrane surface. Furthermore, this formulation demonstrated excellent fouling resistance, maintaining a high flux recovery rate after multiple filtration cycles [95].

#### 4.2.4. Forward Osmosis

Forward osmosis (FO) is a separation process that utilizes the difference in osmotic pressure between a feed solution and a draw solution, which are separated by a semi-permeable membrane. This osmotic potential difference spontaneously drives water from the feed solution to the draw solution, while undesirable solutes remain retained on the feed side [96].

Among the main advantages of FO are its low energy consumption, since the process does not require applied hydraulic pressure, low membrane fouling propensity, and high-water recovery efficiency. These characteristics make the technology especially attractive for wastewater treatment [18]. However, FO also presents limitations, such as the scarcity of ideal draw solutes, challenges in selecting suitable membranes, and issues related to both internal and external concentration polarization [18,97].

As an example of forward osmosis application, ref. [96] developed a membrane based on a continuous layer of Na-ZSM-5 aluminosilicate zeolite crystals, synthesized directly on the outer surface of a porous α-alumina tubular support. This membrane was evaluated for its rejection performance and fouling resistance. The results indicated high rejection efficiency, reaching 99% for various metallic species, including As (III), As(V), Se (IV), Se (VI), and Cr (VI). This high performance was attributed to the molecular sieving effect provided by the zeolite pores and the electrostatic repulsion between the anions present in the solutions and the anionic structure of the membrane.

#### 4.2.5. Ultrafiltration

UF membranes have pore sizes between 5 and 20 nm, suitable for isolating heavy metals, suspended solids, and macromolecules with molecular weights in the range of 10,000 to 100,000 Da [94]. They offer advantages such as lower operating pressure compared to other techniques, reduced space requirements, lower chemical addition, mild operating temperature, and higher permeability than RO and NF [97].

UF can be applied through two techniques specifically developed for the removal of metal ions: micellar-enhanced ultrafiltration (MEUF) and polymer-enhanced ultrafiltration (PEUF) [97]. The MEUF technique is particularly suitable for treating wastewater with low concentrations of heavy metals, being based on the combination of UF membranes with surfactants. This approach offers high permeate flux and high selectivity. However, the recovery of surfactants in the UF concentrate stream and the operational cost still represent technical and economic challenges to be overcome [18,97].

On the other hand, PEUF is formed by integrating UF membranes with binding polymers, aiming to separate heavy metals and dissolved organic compounds in aqueous solutions. This technique has demonstrated effective performance in removing various metal ions, such as Cr^3+^, Pd^2+^, Ni^2+^, Mn^2+^, Cu^2+^, Hg^2+^, and Cd^2+^, and is widely applied in the treatment of industrial effluents contaminated with toxic metals [18,97].

Indeed, to overcome the aforementioned technical and economic challenges, reagent recovery is a critical factor for the industrial viability of both MEUF and PEUF. For MEUF, surfactant recovery is addressed by strategies that include chemical precipitation via acidification and foam fractionation. Analogously, in PEUF, polymers are regenerated by eluting the bound ions using strong acids or ligands, or through electrolysis to promote the electrodeposition of contaminants. The development and optimization of these recovery routes remain a fundamental step for the successful transition of these technologies to an industrial scale [98].

#### 4.2.6. Reverse Osmosis

Membranes used in reverse osmosis (RO) processes can retain particles and solutes with extremely small sizes, ranging from 0.00025 to 0.003 μm. The process operates under high pressure, reversing the natural osmotic flow by forcing water through the semi-permeable membrane from the feed solution to the permeate side. This technology stands out for its remarkable separation capability, being efficient in removing microscopic particles and monovalent ions such as sodium (Na^+^) and chloride (Cl^−^), achieving rejection rates of 95 to 99% for inorganic salts and charged organic compounds [18].

Reverse osmosis has been widely used in water treatment and desalination, accounting for more than 65% of desalination plants in operation worldwide. Technology enables the production of high-purity water, suitable for human consumption, agricultural use, and industrial applications, promoting the efficient removal of salts, minerals, and ions present in the feed water [99].

One of the main challenges in the application of traditional RO membranes is their limited lifespan, often compromised by fouling processes, degradation of the polymeric structure, reduction in salt rejection rate, and decreased water flux as the membrane ages [99]. In response to these limitations, research focused on membrane modification has gained prominence in recent years. Among the most promising strategies is the incorporation of nanomaterials into the PA matrix, aiming to enhance its physicochemical properties. Thin-film composite (TFC) membranes have become the main class used in the manufacture of modified RO membranes, offering significant improvements in separation efficiency, wear resistance, and operational durability [99,100].

#### 4.2.7. Electrodialysis

Electrodialysis is a separation process that uses an electric field as the driving force to promote the migration of ions between solutions through selective ion-exchange membranes [99]. The system consists of a series of cation exchange membranes (CEM) and anion exchange membranes (AEM), alternately arranged in parallel within an electrodialysis stack. During the process, anions pass through the AEMs, while cations migrate through the CEMs, allowing for the separation of ionic solutes. Consequently, half of the channels in the stack produce the diluted stream, while the other half accumulates the concentrated stream [18].

A detailed study conducted by [101] presented a practical example of this process, in which dissolved cations such as K^+^, Ca^2+^, Mg^2+^, and Na^+^, present in feed water (such as seawater or brackish water), migrate toward the cathode (negative electrode) through the CEMs, which selectively allow the passage of positively charged ions. Simultaneously, anions such as Cl^−^, SO_4_^2−^, and NO_3_^−^ are directed to the anode (positive electrode) through the AEMs, which, in turn, are selective for the passage of negatively charged ions.

The CEMs, negatively charged, prevent the passage of anions by electrostatic repulsion, while the AEMs, positively charged, block the migration of cations. In this way, the ion-exchange membranes play an essential role in the precise control of ion movement in electrodialysis, ensuring the efficiency of the separation process [101].

## 5. Results

### 5.1. Data Sources and Methodology

**Figure 5:**
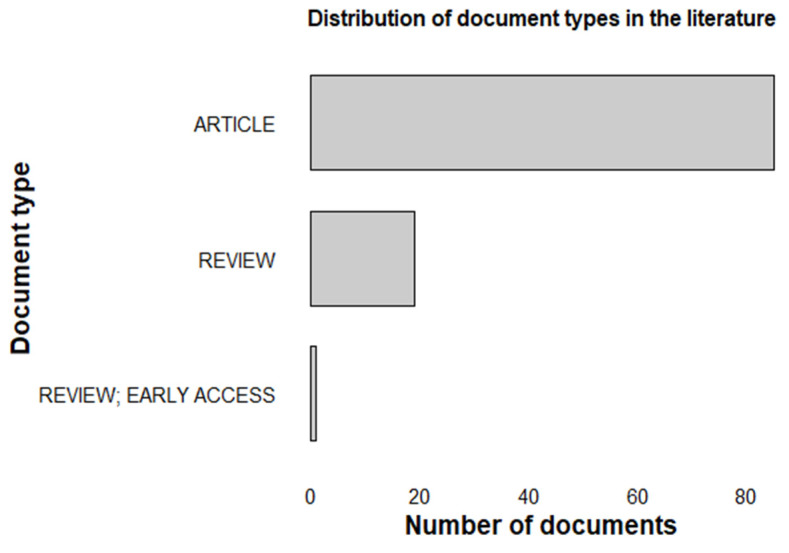
Classification of documents obtained from the literature search on the Web of Science and Scopus platforms.

This bibliometric analysis enabled the mapping and characterization of scientific output related to advanced materials for heavy metal removal, highlighting trends, temporal evolution, and main research topics. The use of the Scopus and Web of Science databases ensured a robust and representative sample, with 120 documents analyzed, covering a period of 2003–2025, including 98 articles, 21 reviews, and 1 early access review, Figure 5.

**Figure 6:**
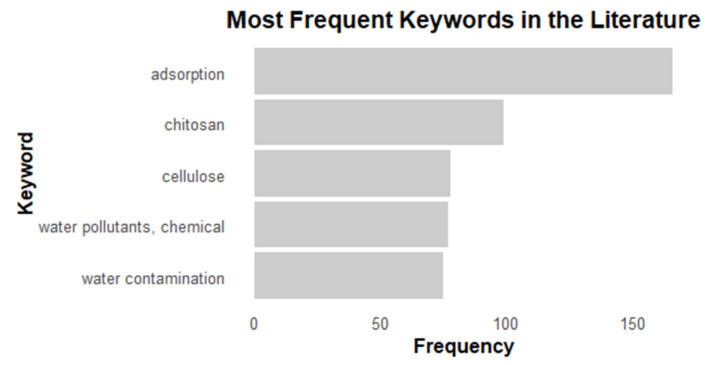
Frequency of occurrence of keywords identified in the bibliographic search. The keywords: adsorption, chitosan, cellulose, water pollutants, chemical and water contamination.

**Figure 7:**
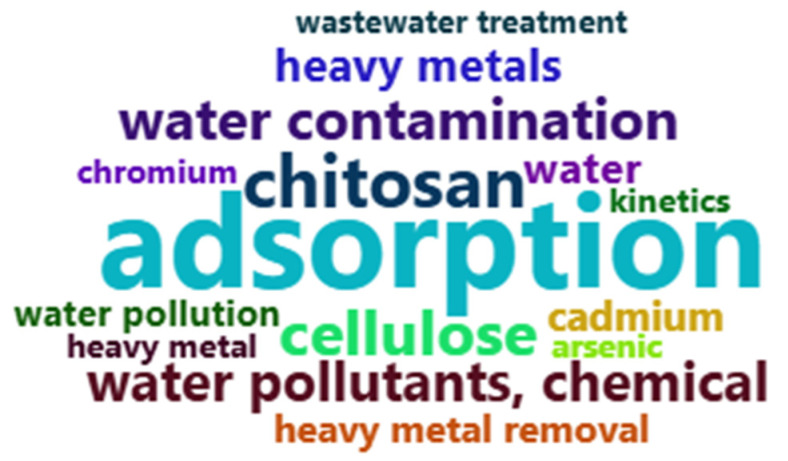
Word cloud of the most searched keywords.

The analysis of keywords revealed the main research focuses in the field. The term “adsorption” appeared most frequently, with 166 occurrences, followed by “chitosan” (99 occurrences), “cellulose” (78 occurrences), “water pollutants, chemical” (77 occurrences), and “water contamination” (75 occurrences). These results indicate that the primary research efforts concentrate on adsorption processes and the use of biopolymeric materials for addressing chemical contamination in water, as shown in Figure 6 and Figure 7

The results demonstrate not only the growth of the field but also the need for continued investment in research and innovation, especially in interdisciplinary approaches focusing on real solutions to environmental challenges.

### 5.2. Adsorptive Capacity

Aerogels and hydrogels derived from biopolymers show significant performance in the adsorption of metal ions from aqueous solution, combining high surface area, the presence of active functional groups, and the possibility of regeneration. Among the materials studied, cellulose aerogels exhibit typical adsorptive capacities between 20 and 200 mg·g^−1^, acting mainly through complexation via hydroxyl (-OH) and carboxyl (-COOH) groups when functionalized. The optimal pH is generally between 5 and 7, and regeneration maintains about 80–95% of capacity after three to five desorption cycles with dilute acids or EDTA [102,103,104].

Chitosan aerogels stand out for their high performance, reaching 100–400 mg·g^−1^, with mechanisms dominated by chelation of metal ions via amino (-NH_2_) and hydroxyl groups. The ideal pH range is around 5–6, and regeneration, usually with dilute HCl or HNO_3_ solutions, maintains 85–98% of the initial capacity for up to five cycles [105,106,107].

Lignin aerogels, including their carbonized versions, show intermediate capacities (50–300 mg·g^−1^), with adsorption based on the complexation of phenolic and carboxylic groups, as well as π-π interactions, especially relevant for dyes. The optimal pH is also concentrated in the 5–7 range, and regeneration efficiency can reach 90–96% after five cycles [108,109].

Alginate-based systems, whether applied as aerogels or hydrogels, exhibit comparable performance (80–300 mg·g^−1^), where the predominant mechanism is ion exchange via the “egg-box” structure and complexation through carboxylate groups. The optimal pH is slightly more acidic (4–6), and regeneration with CaCl_2_ or weak acids maintains 70 to 95% of the initial efficiency [110,111].

More generally, bio-based aerogels can achieve capacities of up to 600 mg·g^−1^ when functionalized for specific ions, combining physical adsorption in nanometric pores with chemical functionalization (-NH_2_, -COOH, -SH). The optimal pH ranges from 4 to 7, and their structural stability allows for more than five regeneration cycles while maintaining 80–95% of the initial capacity [112]. Bio-based hydrogels, in turn, show capacities of 50–300 mg·g^−1^, with performance governed by diffusion and swelling of the polymeric network, in addition to ion exchange mechanisms. They typically operate at pH 4–6 and retain between 70 and 90% efficiency after three to five cycles [60,110]. A comparative summary of these properties is presented in Table 1.

In summary, although all studied biopolymers show potential for environmental applications, materials rich in amino groups, such as chitosan, and those with specific functionalizations stand out for their higher performance and better regeneration, while cellulose, lignin, and alginate offer sustainable alternatives with good efficiencies under controlled conditions. These high-performance materials represent the most recent advances in the field, where biopolymers are engineered into composites or are chemically functionalized to create structures designed specifically to maximize adsorption. Table 2 illustrates these advanced applications with concrete examples, detailing the remarkable adsorption capacities achieved by these next-generation biosorbents.

It is important to note that the adsorption capacities summarized in Table 1 are indicative ranges collected from diverse studies, often performed under different conditions (e.g., solution composition, pH, contact time, isotherm model, real vs. model effluents). Therefore, they should be interpreted as qualitative guidelines rather than direct quantitative comparisons. For rigorous evaluation, Table 2 provides specific case studies with complete experimental conditions and reported adsorption values.

## 6. Practical Applications and Case Studies

The use of sustainable adsorbents has advanced significantly, with recent studies demonstrating their effectiveness in removing heavy metals from industrial effluents. Materials derived from agro-industrial waste, such as coconut shells, stand out for their low cost, wide availability, and regeneration capacity, as well as high efficiency in adsorbing metal ions, including chromium and lead [116]. Analogously, other low-cost carbonaceous materials, such as coal, have also been evaluated as efficient adsorbents for the removal of different types of pollutants, like phosphate, from aqueous effluents [117].

In the electroplating industry, research has investigated the use of iron-doped activated carbon derived from coconut shells for the removal of heavy metals such as hexavalent chromium from industrial effluents. The material showed high adsorption capacity for Cr (VI), and studies indicate that its production cost may be lower than that of commercial synthetic adsorbents. This approach combines technical efficiency with reduced environmental and economic impacts [116].

In the agricultural sector, residues such as rice husks have been used to treat wastewater contaminated with metals. These biosorbents demonstrate the potential of these materials to promote sustainable agriculture. The use of biosorbents derived from agro-industrial waste is relevant for the removal of metals that can contaminate the environment [118]. These successful case studies highlight the potential economic advantages of biosorbents, which merits a broader analysis of their cost–benefit ratio compared to established technologies.

Comparative studies showing the cost–benefit ratio of biosorbents versus polymeric membranes for large-scale wastewater treatment are scarce in the scientific literature. However, the general consensus is that biosorption presents a significant economic advantage, mainly due to the low cost of the materials used [10]. The main reason for the favorable cost–benefit of biosorbents is that they are produced from agricultural or industrial waste, considered low-cost or “zero-cost” materials. In contrast, conventional methods such as membrane techniques are often cited as expensive, involving high operational and capital costs [11,119].

Economic analysis shows that biosorption can reduce capital costs by about 20% and operational costs by 36% compared to conventional systems. While the cost of biosorbents depends on factors such as processing and regeneration potential, the use of waste as a raw material represents a clear economic advantage [120]. On the other hand, the cost of membrane technologies is impacted by the high prices of resins and modules, in addition to the vulnerability to fouling by organics, especially on a large scale [11]. The literature acknowledges that the lack of pilot-scale studies makes a direct numerical comparison difficult, but the current consensus is that the operational simplicity and low cost of materials give biosorbents a significant economic advantage [10].

Despite advances, various regulatory and economic barriers hinder large-scale adoption. Many countries lack specific regulations that recognize and regulate the use of alternative adsorbents, which limits their formal use in industrial processes. Additionally, the initial cost of adapting industrial plants and the absence of tax incentives or subsidies make it difficult for these materials to compete with traditional solutions. In developing countries, technological adoption has advanced through partnerships between universities, research centers, and companies [121].

There are commercial products that fit into the category of sustainable adsorbents, especially those derived from agro-industrial waste. Although the market for these may not be as developed as that for traditional synthetic adsorbents, their potential is significant [121].

## 7. Conclusions

A preliminary study was conducted using data collection from databases on the topic addressed in this work, followed by the use of R software, ensuring that the methods employed can be reproduced and adapted as needed. The bibliometrix package proved to be a valuable tool for organizing and analyzing bibliographic data.

Although studies on multifunctional materials focused on the adsorption of heavy metals have shown significant progress, the analysis revealed some issues that have not yet been thoroughly investigated. These limitations highlight the importance of developing research that combines knowledge from various fields, including materials development, toxicity studies, and sustainable production models.

In this way, advanced materials can become a highly viable alternative for the treatment of polluted waters. Nevertheless, it is essential to focus on experiments with real effluent samples and on improving the removal capacity of various pollutants, aiming also to reduce production and operational costs, and to ensure that the processes are environmentally safe and scalable.

Another aspect that deserves attention is the emergence of new technologies, such as advanced nanomaterials and methods for treating multiple contaminants simultaneously, which have not yet been sufficiently investigated. Future studies aim to focus on making these solutions financially viable, testing them in real-world situations, and incorporating computational simulation techniques to optimize their performance.

Furthermore, there are still possibilities to improve certain parameters.

1. The practical application of sustainable adsorbents for adoption on industrial scale.
2. The sustainability of the synthesis methods for these materials.
3. These materials need to be integrated with the emerging technologies.
4. These adsorbents can be combined with sensors for real-time monitoring.
5. Adequate infrastructure and qualified manpower are to be developed for these adsorbent materials.
6. Low selectivity, loss of efficiency after a few regeneration cycles, and difficulty in application to complex effluents with multiple contaminants.
7. Adsorbents which have greater chemical stability and which can be reused without compromising the performance still need to be developed.
8. The disposal of these adsorbents after their useful life is a problem that needs to be addressed.
9. There is still lack of regulation for ensuring the safety of environment in the use and reuse of adsorbents which needs to be considered.
10. Standardized and efficient synthesis routes must be utilized to overcome certain limitations.

## Figures and Tables

**Table 1:** Comparison of the adsorptive and operational properties of biopolymer aerogels and hydrogels in the removal of metal ions from aqueous solution.

Material	Adsorption Capacity for Metals	Predominant Mechanisms	Optimum pH for Metals	Regeneration Efficiency ^1^	References
Cellulose	20–200	Complexation by -OH (and -COOH/-NH_2_ after functionalization), electrostatic interactions; diffusion in pores	5–7	80–95% of capacity after 3–5 cycles (diluted acids/EDTA)	[102,103,104]
Chitosan	100–400	Chelation via -NH_2_/-OH, ion exchange, and electrostatic interactions; strong affinity for Pb^2+^, Cu^2+^	5–6	85–98% after 3–5 cycles (diluted HCl/HNO_3_), depending on modification	[105,106,107]
Lignin	50–300	Complexation via phenolic -OH/-COOH, π-interactions (for dyes), electrostatics	5–7	~90–96% after up to 5 reported cycles	[108,109]
Alginate	80–300	Ion exchange (egg-box structure), complexation by -COO^−^; occasional cross-linking with Ca^2+^	4–6	70–95% after 3–5 cycles (CaCl_2_/weak acids)	[110,111]
Bio-based aerogels	50–600 ^2^	Physical adsorption in high specific area + functional groups (-NH_2_, -COOH, -SH, etc.)	4–7	>80–95% for 5–8 cycles in many studies	[112]
Bio-based hydrogels	50–300	Ion exchange, chelation; controlled uptake by swelling/diffusion	4–6	70–90% after 3–5 cycles (depending on crosslinker)	[60,110]

^1^ Regeneration efficiency expressed as retention of capacity after adsorption–desorption cycles with common eluents (dilute acids/saline solutions). ^2^ The upper limit (≈600 mg·g^−1^) appears in highly functionalized aerogels for specific metals; “typical” values are lower.

**Table 2:** Adsorption capacity of specific biopolymeric adsorbents for the removal of heavy metal ions.

Biopolymer Type	Specific Adsorbent Material	Metal Ion	Adsorption Capacity (mg·g^−1^)	References
Cellulose	Spherical-shaped graphene oxide-embedded chitosan/gelatin hydrogel	Cd^2+^	126.58	[110]
	Polyethyleneimine (PEI)-grafted adsorbent, a cellulose@PEI aerogel (CPA-2)	Cr^6+^	96.8	[102]
	C6 carboxylic microcrystalline cellulose	Cu^2+^	165.5	[41]
	Salix psammophila cellulose aerogel	Cu^2+^, Mn^2+^ and Zn^2+^	272.69, 253.25 and 143.00	[41]
	Bacterial cellulose graphene oxide composite	Pb^2+^	116.54	[102]
	TEMPO-oxidized (TO) nanofibrillated cellulose (TO-NFC) aerogel	Hg^2+^	140.25	[102]
Chitosan	Polyethyleneimine modified carboxymethyl chitosan aerogel	Cu^2+^	175.56	[106]
	Bentonite-modified chitosan/microcrystalline cellulose aerogel-prepared	Pb^2+^	116.54	[102]
	Gallic acid modified carboxymethyl chitosan/iron ion complex hydrogels	Pb^2+^, Cd^2+^ and Cu^2+^	97.15, 99.75 and 98.50	[106]
	Magnetic mesoporous silica/chitosan (MMS/CS)	Hg^2+^	478.47	[113]
	Polydopamine functionalized graphene oxide/carboxymethyl chitosan composite aerogels	Cu^2+^, Ni^2+^ and Pb^2^	170.3, 186.8 and 312.8	[82]
Lignin	Fluorescent lignin-based hydrogel with cellulose nanofibers and carbon dots	Cr6^+^	599.9	[86]
	Organosolv Lignin with H3[PMo12O40](POM)	Cd^2+^ and Pb^2+^	35.9 and 155.4	[114]
	Polyethyleneimine functionalized chitosan–lignin (PEI-CS-L)	Hg^2+^	663.5	[114]
	Poly (ethylene imine) anchored lignin composite	Cu^2+^	98	[114]
Alginate	Magnetic sodium alginate/carboxymethyl cellulose	Mn^2+^, Pb^2+^ and Cu^2+^	71.83, 89.49 and 105.93	[86]
	Phosphate-embedded calcium alginate beads	Pb^2+^ and Cd^2+^	263.16 and 82.64	[115]
	Calcium alginate/carboxymethylated chitosan/Na-bentonite	Ni^2+^	159	[86]
	Calcium-alginate immobilized-algal beads	Hg^2+^	116.8	[115]
	Sodium alginate/polyethyleneimine composite hydrogel	Cu^2+^ and Pb^2+^	322.6 and 344.8	[86]

## Data Availability

Not applicable.

## References

[1] Lubal M.J. (2024). Health Effects of Heavy Metal Contamination in Drinking Water. UTTAR PRADESH J. Zool..

[2] Manzini F.F., Sá K.B.d., de Almeida Plicas L.M. (2010). Metais Pesados: Fonte E Ação Toxicológica. Periódico Eletrônico Fórum Ambient. Da Alta Paul..

[3] Ellwanger J.H., Chies J.A.B. (2023). Brazil’s Heavy Metal Pollution Harms Humans and Ecosystems. Sci. One Health.

[4] Taing L. (2022). Is Safe Water, Sanitation, and Hygiene a Pipe Dream?. One Earth.

[5] Dahiya V. (2022). Heavy Metal Toxicity of Drinking Water: A Silent Killer. GSC Biol. Pharm. Sci..

[6] Nascimento R.F.d., Sousa Neto V.d.O., Melo D.d.Q. (2014). Uso de Bioadsorventes Lignocelulósicos na Remoção de Poluentes de Efluentes Aquosos. https://www.researchgate.net/publication/308349576_Uso_de_bioadsorventes_lignocelulosicos_na_remocao_de_poluentes_de_efluentes_aquosos.

[7] Reis J.M.d., Aguiar A.B.S., Freitas G., Vassoler V.C., Barros G.V.L., Santos G.E., Ramirez I., Rodriguez R.P. (2022). Técnicas de remoção de metais de águas residuárias: Uma revisão de literatura. Res. Soc. Dev..

[8] Figoli A., Marino T., Galiano F., Dorraji S.S., Di Nicolò E., He T., Figoli A., Criscuoli A. (2017). Sustainable Route in Preparation of Polymeric Membranes. Sustainable Membrane Technology for Water and Wastewater Treatment.

[9] Loh C.Y., Burrows A.D., Xie M. (2025). Sustainable Polymeric Membranes: Green Chemistry and Circular Economy Approaches. ACS EST Eng..

[10] Younas F., Mustafa A., Farooqi Z.U.R., Wang X., Younas S., Mohy-Ud-Din W., Ashir Hameed M., Mohsin Abrar M., Maitlo A.A., Noreen S. (2021). Current and Emerging Adsorbent Technologies for Wastewater Treatment: Trends, Limitations, and Environmental Implications. Water.

[11] Davoodbeygi Y., Askari M., Salehi E., Kheirieh S. (2025). A Review on Hybrid Membrane-Adsorption Systems for Intensified Water and Wastewater Treatment: Process Configurations, Separation Targets, and Materials Applied|Request PDF. ResearchGate.

[12] Sabando-Fraile C., Corral-Bobadilla M., Lostado-Lorza R., Gallarta-González F. (2024). Applying Circular Economy Principles and Life Cycle Assessment: A Novel Approach Using Vine Shoots Waste for Cadmium Removal from Water. Sci. Total Environ..

[13] Overview—SDG Indicators. https://unstats.un.org/sdgs/report/2021/Overview.

[14] Posit Download RStudio Server v2025.09.0+387. https://posit.co/download/rstudio-server/.

[15] Posit RStudio Desktop. https://posit.co/download/rstudio-desktop/.

[16] Zaimee M.Z.A., Sarjadi M.S., Rahman M.L. (2021). Heavy Metals Removal from Water by Efficient Adsorbents. Water.

[17] Singh V., Ahmed G., Vedika S., Kumar P., Chaturvedi S.K., Rai S.N., Vamanu E., Kumar A. (2024). Toxic Heavy Metal Ions Contamination in Water and Their Sustainable Reduction by Eco-Friendly Methods: Isotherms, Thermodynamics and Kinetics Study. Sci. Rep..

[18] Qasem N.A.A., Mohammed R.H., Lawal D.U. (2021). Removal of Heavy Metal Ions from Wastewater: A Comprehensive and Critical Review. npj Clean Water.

[19] Velusamy S., Roy A., Sundaram S., Kumar Mallick T. (2021). A Review on Heavy Metal Ions and Containing Dyes Removal Through Graphene Oxide-Based Adsorption Strategies for Textile Wastewater Treatment. Chem. Rec..

[20] Fei Y., Hu Y.H. (2023). Recent Progress in Removal of Heavy Metals from Wastewater: A Comprehensive Review. Chemosphere.

[21] Punia P., Bharti M.K., Dhar R., Thakur P., Thakur A. (2022). Recent Advances in Detection and Removal of Heavy Metals from Contaminated Water. ChemBioEng Rev..

[22] Zhu Y., Fan W., Zhou T., Li X. (2019). Removal of Chelated Heavy Metals from Aqueous Solution: A Review of Current Methods and Mechanisms. Sci. Total Environ..

[23] Razzak S.A., Faruque M.O., Alsheikh Z., Alsheikhmohamad L., Alkuroud D., Alfayez A., Hossain S.M.Z., Hossain M.M. (2022). A Comprehensive Review on Conventional and Biological-Driven Heavy Metals Removal from Industrial Wastewater. Environ. Adv..

[24] Bashir A., Malik L.A., Ahad S., Manzoor T., Bhat M.A., Dar G.N., Pandith A.H. (2019). Removal of Heavy Metal Ions from Aqueous System by Ion-Exchange and Biosorption Methods. Environ. Chem. Lett..

[25] Yang S.-C., Liao Y., Karthikeyan K.G., Pan X.J. (2021). Mesoporous Cellulose-Chitosan Composite Hydrogel Fabricated via the Co-Dissolution-Regeneration Process as Biosorbent of Heavy Metals. Environ. Pollut..

[26] Doyo A.N., Kumar R., Barakat M.A. (2023). Recent Advances in Cellulose, Chitosan, and Alginate Based Biopolymeric Composites for Adsorption of Heavy Metals from Wastewater. J. Taiwan Inst. Chem. Eng..

[27] Ihsanullah I., Sajid M., Khan S., Bilal M. (2022). Aerogel-Based Adsorbents as Emerging Materials for the Removal of Heavy Metals from Water: Progress, Challenges, and Prospects. Sep. Purif. Technol..

[28] Zhao J., Li H., Mu C., Zhang S., Shi F., Hu J. (2024). Performance and Mechanism of L-Arginine Modifed Alginate Aerogels for Adsorption of Cadmium and Copper Ions. J. Polym. Environ..

[29] Zhang Y., Luo J., Zhang H., Li T., Xu H., Sun Y., Gu X., Hu X., Gao B. (2022). Synthesis and Adsorption Performance of Three-Dimensional Gels Assembled by Carbon Nanomaterials for Heavy Metal Removal from Water: A Review. Sci. Total Environ..

[30] Mirbagheri S., Kalantari M., Miroliaei M.R. (2025). Optimization of a Novel Waste-Based Hydrogel and Its Application in Heavy Metal Removal. Polym. Bull..

[31] Tang R., Zhang H., Muhammad Y., Lu C., Liu K., Yu S., Tong Z. (2022). Preparation of Polyethylenimine and Carboxymethyl Cellulose Co-Modified Magnetic Bentonite for Enhanced Adsorption of Pb(II) and Cd(II) Based on the Concept of Mesh Bag and Octopus-like Tentacle. Cellulose.

[32] Zheng D., Wang K., Bai B. (2024). A Critical Review of Sodium Alginate-Based Composites in Water Treatment. Carbohydr. Polym..

[33] Wei G., Miao Y.-E., Zhang C., Yang Z., Liu Z., Tjiu W.W., Liu T. (2013). Ni-Doped Graphene/Carbon Cryogels and Their Applications As Versatile Sorbents for Water Purification. ACS Appl. Mater. Interfaces.

[34] Zhan W., Gao L., Fu X., Siyal S.H., Sui G., Yang X. (2019). Green Synthesis of Amino-Functionalized Carbon Nanotube-Graphene Hybrid Aerogels for High Performance Heavy Metal Ions Removal. Appl. Surf. Sci..

[35] Yang T., Xu Y., Huang Q., Sun Y., Liang X., Wang L., Qin X., Zhao L. (2021). Adsorption Characteristics and the Removal Mechanism of Two Novel Fe-Zn Composite Modified Biochar for Cd(II) in Water. Bioresour. Technol..

[36] Gökırmak Söğüt E., Gülcan M., Verma C., Aslam J., Khan M.E. (2023). Chapter 1—Adsorption: Basics, Properties, and Classification. Adsorption through Advanced Nanoscale Materials.

[37] Neethu T.M., Dubey P.K., Patel K.G., Nilima K., Aakash M. (2023). Bio-Sorbents: A Novel Technology to Mitigate Heavy Metal Pollution. Int. J. Plant Soil Sci..

[38] Xiang H., Min X., Tang C.-J., Sillanpää M., Zhao F. (2022). Recent Advances in Membrane Filtration for Heavy Metal Removal from Wastewater: A Mini Review. J. Water Process Eng..

[39] Niculescu A.-G., Tudorache D.-I., Bocioagă M., Mihaiescu D.E., Hadibarata T., Grumezescu A.M. (2024). An Updated Overview of Silica Aerogel-Based Nanomaterials. Nanomaterials.

[40] Li L., Jin G., Shen J., Guo M., Song J., Li Y., Xiong J. (2025). Carbon Aerogels: Synthesis, Modification, and Multifunctional Applications. Gels.

[41] Zhong Y., An Y., Wang K., Zhang W., Hu Z., Chen Z., Wang S., Wang B., Wang X., Li X. (2022). Evaluation of Aerogel Spheres Derived from Salix Psammophila in Removal of Heavy Metal Ions in Aqueous Solution. Forests.

[42] Garg S., Singh S., Shehata N., Sharma H., Samuel J., Khan N.A., Ramamurthy P.C., Singh J., Mubashir M., Bokhari A. (2025). Aerogels in Wastewater Treatment: A Review. J. Taiwan Inst. Chem. Eng..

[43] Zhong Y., Qin L., Wang X., Liu W., Yang Y., Liu X. (2025). Carbon Aerogel for Aqueous Phase Adsorption/Absorption: Application Performances, Intrinsic Characteristics, and Regulatory Constructions. Small Struct..

[44] Suryapet P., Allaparthi M., Chary T.R.G. (2024). Intelligent Manufacturing and Energy Sustainability.

[45] Zhou W., Deng J., Qin Z., Huang R., Wang Y., Tong S. (2022). Construction of MoS2 Nanoarrays and MoO3 Nanobelts: Two Efficient Adsorbents for Removal of Pb(II), Au(III) and Methylene Blue. J. Environ. Sci..

[46] Basu A., Ali S.S., Hossain S.S., Asif M. (2022). A Review of the Dynamic Mathematical Modeling of Heavy Metal Removal with the Biosorption Process. Processes.

[47] Shrestha R., Ban S., Devkota S., Sharma S., Joshi R., Tiwari A.P., Kim H.Y., Joshi M.K. (2021). Technological Trends in Heavy Metals Removal from Industrial Wastewater: A Review. J. Environ. Chem. Eng..

[48] Wang F., Lu X., Li X. (2016). Selective Removals of Heavy Metals (Pb2+, Cu2+, and Cd2+) from Wastewater by Gelation with Alginate for Effective Metal Recovery. J. Hazard. Mater..

[49] Vasil’eva V.I., Meshcheryakova E.E., Chernyshova O.I., Brovkina M.A., Falina I.V., Akberova E.M., Dobryden’ S.V. (2024). Transport and Structural Characteristics of Heterogeneous Ion-Exchange Membranes with Varied Dispersity of the Ion Exchanger. Membr. Membr. Technol..

[50] De Torres Vincent T., Boyer T.H. (2020). Beneficial Reuse of Treated Municipal Wastewater and Flue Gas Carbon Dioxide via Combined Ion Exchange. J. Water Process Eng..

[51] Hasanpour M., Hatami M. (2020). Application of Three Dimensional Porous Aerogels as Adsorbent for Removal of Heavy Metal Ions from Water/Wastewater: A Review Study. Adv. Colloid Interface Sci..

[52] Arora R. (2019). Adsorption of Heavy Metals–A Review. Mater. Today Proc..

[53] Gusain D., Bux F., Gusain D., Bux F. (2021). Batch Adsorption Process of Metals and Anions for Remediation of Contaminated Water.

[54] Daochalermwong A., Chanka N., Songsrirote K., Dittanet P., Niamnuy C., Seubsai A. (2020). Removal of Heavy Metal Ions Using Modified Celluloses Prepared from Pineapple Leaf Fiber. ACS Omega.

[55] Baskar A.V., Bolan N., Hoang S.A., Sooriyakumar P., Kumar M., Singh L., Jasemizad T., Padhye L.P., Singh G., Vinu A. (2022). Recovery, Regeneration and Sustainable Management of Spent Adsorbents from Wastewater Treatment Streams: A Review. Sci. Total Environ..

[56] Tariq A., Yahaya N., Sajid M. (2024). Low Cost Adsorbents Derived from Vegetables and Fruits: Synthesis, Properties, and Applications in Removal of Heavy Metals from Water. Desalination Water Treat..

[57] Boyle O., Xiao B., Mangwandi C. (2025). Valorization of Banana Peel Waste into Advanced Adsorbent Beads for the Removal of Emerging Pollutants from Wastewater. Materials.

[58] Akram M., Bano Z., Bhutto S.U.A., Pan J., Majeed M.K., Li L., Xia M., Wang F. (2025). Sustainable Synthesis of Ce-La Loaded onto *Citrus Limon* for Phosphate Removal: Performance and Regeneration Studies. Colloids Surf. B Biointerfaces.

[59] Taha A.M., Mustafa F.H.A., Ibrahim H.E., Mohamadein L.I., Anwar Z.M., Elsharaawy R.F.M. (2025). Adsorptive Removal of Heavy Metal Ions from Wastewater Using Shrimp Chitosan-Cysteine-Glutaraldehyde Hydrogel as a Sustainable Biosorbent. Int. J. Biol. Macromol..

[60] Mohanrasu K., Manivannan A.C., Rengarajan H.J.R., Kandaiah R., Ravindran A., Panneerselvan L., Palanisami T., Sathish C.I. (2025). Eco-Friendly Biopolymers and Composites: A Sustainable Development of Adsorbents for the Removal of Pollutants from Wastewater. npj Mater. Sustain..

[61] Yulia R., Husin H., Zaki M., Razali N., Hisbullah, Nasution F., Ahmadi, Nurhazanah, Yazil M.L., Sy Y. (2025). Enhancing Sustainability through Optimized Adsorption Using a Novel Klason-Lignin-Based Biosorbent Derived from Sugar-Palm Fruit Shells for Efficient Removal of Pb(II) and Cd(II). Energy Nexus.

[62] Fouda-Mbanga B.G., Prabakaran E., Pillay K. (2021). Carbohydrate Biopolymers, Lignin Based Adsorbents for Removal of Heavy Metals (Cd2+, Pb2+, Zn2+) from Wastewater, Regeneration and Reuse for Spent Adsorbents Including Latent Fingerprint Detection: A Review. Biotechnol. Rep..

[63] Trivunac K., Mihajlović S., Vukčević M., Maletić M., Pejić B., Kalijadis A., Perić Grujić A. (2024). Modified Cellulose-Based Waste for Enhanced Adsorption of Selected Heavy Metals from Wastewater. Polymers.

[64] Barzegarzadeh M., Hazrati A., Amini-Fazl M.S. (2025). Cellulose Extraction from Corn Husk for Cellulose-Based Bionanocomposite Preparation with Remarkable Adsorption Capacity for Doxorubicin Drug: Emphasis on Effects Ultrasonic Waves. Int. J. Biol. Macromol..

[65] Freitas P.A.V., Collado P.A., González-Martínez C., Chiralt A. (2025). Producing Aerogels from Rice Straw Cellulose Obtained by a Green Method and Its Starch Blending. Polymers.

[66] Ma Y., Hu Y., Yang X., Shang Q., Huang Q., Hu L., Jia P., Zhou Y. (2025). Fabrication, Functionalization and Applications of Cellulose Based Aerogels: A Review. Int. J. Biol. Macromol..

[67] Alatawi I.S.S., Almughathawi R., Madkhali M.M.M., Alshammari N.M., Alaysuy O., Mogharbel A.T., Hosni M., El-Metwaly N.M. (2025). Sustainable Waste-Derived Cellulose-Based Nanosensor for Cobalt Ion Detection, Removal, and Recovery from Industrial Effluents and Battery Wastes. J. Water Process Eng..

[68] Li J., Tan S., Xu Z. (2020). Anisotropic Nanocellulose Aerogel Loaded with Modified UiO-66 as Efficient Adsorbent for Heavy Metal Ions Removal. Nanomaterials.

[69] Gonçalves J.O., Strieder M.M., Silva L.F.O., dos Reis G.S., Dotto G.L. (2024). Advanced Technologies in Water Treatment: Chitosan and Its Modifications as Effective Agents in the Adsorption of Contaminants. Int. J. Biol. Macromol..

[70] Aranaz I., Alcántara A.R., Civera M.C., Arias C., Elorza B., Heras Caballero A., Acosta N. (2021). Chitosan: An Overview of Its Properties and Applications. Polymers.

[71] Atangana E., Ajiboye T.O., Mafolasire A.A., Ghosh S., Hakeem B. (2025). Adsorption of Organic Pollutants from Wastewater Using Chitosan-Based Adsorbents. Polymers.

[72] Miron A., Iordache T.-V., Valente A.J.M., Durães L.M.R., Sarbu A., Ivan G.R., Zaharia A., Sandu T., Iovu H., Chiriac A.-L. (2024). Chitosan-Based Beads Incorporating Inorganic–Organic Composites for Copper Ion Retention in Aqueous Solutions. Int. J. Mol. Sci..

[73] Rahman M.d.H., Marufuzzaman M.d., Rahman M.d.A., Mondal M.d.I.H. (2025). Adsorption Kinetics and Mechanisms of Nano Chitosan Coated Cotton Fiber for the Removal of Heavy Metals from Industrial Effluents. Heliyon.

[74] Camargos C.H.M., Yang L., Jackson J.C., Tanganini I.C., Francisco K.R., Ceccato-Antonini S.R., Rezende C.A., Faria A.F. (2025). Lignin and Nanolignin: Next-Generation Sustainable Materials for Water Treatment. ACS Appl. Bio Mater..

[75] Gupta A., Sharma V., Sharma K., Kumar V., Choudhary S., Mankotia P., Kumar B., Mishra H., Moulick A., Ekielski A. (2021). A Review of Adsorbents for Heavy Metal Decontamination: Growing Approach to Wastewater Treatment. Materials.

[76] Agüero L., Zaldivar-Silva D., Peña L., Dias M.L. (2017). Alginate Microparticles as Oral Colon Drug Delivery Device: A Review. Carbohydr. Polym..

[77] Siddiqui V.U., Ilyas R.A., Sapuan S.M., Hamid N.H.A., Khoo P.S., Chowdhury A., Atikah M.S.N., Rani M.S.A., Asyraf M.R.M. (2025). Alginate-Based Materials as Adsorbent for Sustainable Water Treatment. Int. J. Biol. Macromol..

[78] Kumar B., Singh N., Kumar P. (2024). A Review on Sources, Modification Techniques, Properties and Potential Applications of Alginate-Based Modified Polymers. Eur. Polym. J..

[79] Pandey S., Pande P.P., Chaurasiya A., Kashaudhan K., Chaudhary A. (2025). Development of Eco-Friendly Sodium Alginate-Based Hydrogel for the Effective Removal of Copper(II) and Cobalt(II) from Contaminated Water. Colloid Polym. Sci..

[80] Nassar A.A., Mubarak M.F., El-Sawaf A.K., Zayed M.A., Hemdan M. (2025). Efficient Lead Ion Removal from Aqueous Solutions for Wastewater Treatment Using a Novel Cross-Linked Alginate-Rice Husk Ash-Graphene Oxide-Chitosan Nanocomposite. Int. J. Biol. Macromol..

[81] Nguyen P.T.T., Do N.H.N., Goh X.Y., Goh C.J., Ong R.H., Le P.K., Phan-Thien N., Duong H.M. (2022). Recent Progresses in Eco-Friendly Fabrication and Applications of Sustainable Aerogels from Various Waste Materials. Waste Biomass Valorization.

[82] Sehaqui H., Salajková M., Zhou Q., Berglund L.A. (2010). Mechanical Performance Tailoring of Tough Ultra-High Porosity Foams Prepared from Cellulose I Nanofiber Suspensions. Soft Matter.

[83] Zaman A., Huang F., Jiang M., Wei W., Zhou Z. (2020). Preparation, Properties, and Applications of Natural Cellulosic Aerogels: A Review. Energy Built Environ..

[84] Guo Y., Shen K., Chen Z., Chen S., Wu C., Wu Q., Liu A., Yang P., Ma Z., Yang L. (2025). 3D-Printed Fumed Silica/Sodium Alginate Aerogels for Thermal Insulation. Ceram. Int..

[85] Abou Taleb M.F., Alzidan K. (2024). Multifunctional Applications of Seaweed Extract-Infused Hydroxyethyl Cellulose-Polyvinylpyrrolidone Aerogels: Antibacterial, and Antibiofilm Proficiency for Water Decontamination. Int. J. Biol. Macromol..

[86] Merillas B., Rodríguez-Pérez M.Á., Durães L. (2025). Enhanced Copper-Adsorption Removal from Water by Easy-Handling Silica Aerogel-Polyurethane Foam Composites. J. Ind. Eng. Chem..

[87] Demir A., Kaya M. (2025). Fabrication and Characterization of Bio-Aerogel from Mandarin Peel-Based Agricultural Waste. Fibers Polym..

[88] Castro-Muñoz R., González-Melgoza L.L., García-Depraect O. (2021). Ongoing Progress on Novel Nanocomposite Membranes for the Separation of Heavy Metals from Contaminated Water. Chemosphere.

[89] Teow Y.H., Chong M.T., Ho K.C., Mohammad A.W. (2021). Comparative Environmental Impact Evaluation Using Life Cycle Assessment Approach: A Case Study of Integrated Membrane-Filtration System for the Treatment of Aerobically-Digested Palm Oil Mill Effluent. Sustain. Environ. Res..

[90] Turhan Kara I., Kiyak B., Colak Gunes N., Yucel S. (2024). Life Cycle Assessment of Aerogels: A Critical Review. J. Sol-Gel Sci. Technol..

[91] Maroufi N., Hajilary N. (2023). Nanofiltration Membranes Types and Application in Water Treatment: A Review. Sustain. Water Resour. Manag..

[92] Mahmoud A.E.D., Mostafa E. (2023). Nanofiltration Membranes for the Removal of Heavy Metals from Aqueous Solutions: Preparations and Applications. Membranes.

[93] Sahu L.R., Yadav D., Ingole P.G. (2025). Recent Developments and Innovations in Thin-Film Nanocomposite Nanofiltration: The next-Generation Selective Membrane for Heavy Metal Ion Removal from Water. Chem. Eng. J..

[94] Sandu T., Sârbu A., Căprărescu S., Stoica E.-B., Iordache T.-V., Chiriac A.-L. (2022). Polymer Membranes as Innovative Means of Quality Restoring for Wastewater Bearing Heavy Metals. Membranes.

[95] Sadek S.A., Al-Jubouri S.M. (2024). Highly Efficient Oil-in-Water Emulsion Separation Based on Innovative Stannic Oxide/Polyvinylchloride (SnO_2_/PVC) Microfiltration Membranes. J. Ind. Eng. Chem..

[96] Sakai M., Negishi E., Matsukata M. (2025). Rejection of Heavy Metal Ions in Water by Zeolite Forward Osmosis Membrane. Sep. Purif. Technol..

[97] Qiu F., Chen R., Chung T.-S., Ge Q. (2022). Forward Osmosis for Heavy Metal Removal: Multi-Charged Metallic Complexes as Draw Solutes. Desalination.

[98] Qiu Y., Depuydt S., Ren L.-F., Zhong C., Wu C., Shao J., Xia L., Zhao Y., Van der Bruggen B. (2023). Progress of Ultrafiltration-Based Technology in Ion Removal and Recovery: Enhanced Membranes and Integrated Processes. ACS EST Water.

[99] Ahmed M.A., Mahmoud S.A., Mohamed A.A. (2024). Nanomaterials-Modified Reverse Osmosis Membranes: A Comprehensive Review. RSC Adv..

[100] Zhao D.L., Japip S., Zhang Y., Weber M., Maletzko C., Chung T.-S. (2020). Emerging Thin-Film Nanocomposite (TFN) Membranes for Reverse Osmosis: A Review. Water Res..

[101] Salsabila N., Biçer Y. (2025). Application of Monovalent Selective Membranes and Bipolar Membranes in Electrodialysis: A Review. J. Environ. Chem. Eng..

[102] Ahmad A., Kamaruddin M.A., Abdul Khalil H.P.S., Yahya E.B., Muhammad S., Rizal S., Ahmad M.I., Surya I., Abdullah C.K. (2023). Recent Advances in Nanocellulose Aerogels for Efficient Heavy Metal and Dye Removal. Gels.

[103] Stark F.W., Thue P.S., Missio A.L., Machado F.M., Delucis R.d.A., Andreazza R. (2025). Cellulose-Based Aerogels for Environmentally Sustainable Applications: A Review of the Production, Modification, and Sorption of Environmental Contaminants. Polymers.

[104] Zhao T., Chen Z., Xiao W., Zhou Y., Zhan B., Lyu Y., Li S., Liu Y. (2025). Cellulose Aerogels in Water Pollution Treatment: Preparation, Applications and Mechanism. Adv. Bionics.

[105] Fan S., Chen J., Fan C., Chen G., Liu S., Zhou H., Liu R., Zhang Y., Hu H., Huang Z. (2021). Fabrication of a CO2-Responsive Chitosan Aerogel as an Effective Adsorbent for the Adsorption and Desorption of Heavy Metal Ions. J. Hazard. Mater..

[106] Paul J., Qamar A., Ahankari S.S., Thomas S., Dufresne A. (2024). Chitosan-Based Aerogels: A New Paradigm of Advanced Green Materials for Remediation of Contaminated Water. Carbohydr. Polym..

[107] Tsauria Q.D., Gareso P.L., Tahir D. (2025). Systematic Review of Chitosan-Based Adsorbents for Heavy Metal and Dye Remediation. Integr. Environ. Assess. Manag..

[108] Wu C.-W., Li P.-H., Wei Y.-M., Yang C., Wu W.-J. (2022). Review on the Preparation and Application of Lignin-Based Carbon Aerogels. RSC Adv..

[109] Dong X., Zhang Y., Shao S., Li H., Yan X. (2025). Application of Lignin-Derived Carbon Materials in Adsorption and Separation. Separations.

[110] Zhao C., Liu G., Tan Q., Gao M., Chen G., Huang X., Xu X., Li L., Wang J., Zhang Y. (2023). Polysaccharide-Based Biopolymer Hydrogels for Heavy Metal Detection and Adsorption. J. Adv. Res..

[111] Ling Felicia W.X., Rovina K., Supri S., Matanjun P., Mohd Amin S.F., Abdul Rahman M.N. (2025). Next-Generation Sodium Alginate Hydrogels for Heavy Metal Ion Removal: Properties, Dynamic Adsorption–Desorption Mechanisms, and Sustainable Application Potential. Polym. Bull..

[112] Croitoru A.-M., Niculescu A.-G., Bîrcă A.C., Mihaiescu D.E., Rădulescu M., Grumezescu A.M. (2025). Nanostructured Aerogels for Water Decontamination: Advances, Challenges, and Future Perspectives. Nanomaterials.

[113] He H., Meng X., Yue Q., Yin W., Gao Y., Fang P., Shen L. (2021). Thiol-Ene Click Chemistry Synthesis of a Novel Magnetic Mesoporous Silica/Chitosan Composite for Selective Hg(II) Capture and High Catalytic Activity of Spent Hg(II) Adsorbent. Chem. Eng. J..

[114] Ruthran V.B., Barman P., Kadam R., Kumar A. (2022). Lignin-Based Adsorbent for Effective Removal of Toxic Heavy Metals from Wastewater. Emergent Mater..

[115] Wang B., Wan Y., Zheng Y., Lee X., Liu T., Yu Z., Huang J., Ok Y.S., Chen J., Gao B. (2019). Alginate-Based Composites for Environmental Applications: A Critical Review. Crit. Rev. Environ. Sci. Technol..

[116] Jaber L., Ihsanullah I., Almanassra I.W., Backer S.N., Abushawish A., Khalil A.K.A., Alawadhi H., Shanableh A., Atieh M.A. (2022). Adsorptive Removal of Lead and Chromate Ions from Water by Using Iron-Doped Granular Activated Carbon Obtained from Coconut Shells. Sustainability.

[117] Khan S., Ishaq M., Ahmad I., Hussain S., Ullah H. (2013). Evaluation of Coal as Adsorbent for Phosphate Removal. Arab J Geosci.

[118] Shahid M., Khalid S., ALOthman Z.A., Al-Kahtani A.A., Bibi I., Naz R., Natasha N., Niazi N.K., Iqbal J., Han C. (2025). Trace Element Removal from Wastewater by Agricultural Biowastes: A Data Analysis on Removal Efficacy and Optimized Conditions. Sci. Total Environ..

[119] Georgin J., Meili L., Franco D. (2023). A Review of the Application of Low-Cost Adsorbents as an Alternative Method for Biosorption of Contaminants Present in Water. Lat. Am. Dev. Energy Eng..

[120] Fertu D.I., Bulgariu L., Gavrilescu M. (2022). Modeling and Optimization of Heavy Metals Biosorption by Low-Cost Sorbents Using Response Surface Methodology. Processes.

[121] Liu Q., Yang L., Yang M. (2021). Digitalisation for Water Sustainability: Barriers to Implementing Circular Economy in Smart Water Management. Sustainability.

